# Characterization of gene expression profiles in HBV-related liver fibrosis patients and identification of ITGBL1 as a key regulator of fibrogenesis

**DOI:** 10.1038/srep43446

**Published:** 2017-03-06

**Authors:** Mingjie Wang, Qiming Gong, Jiming Zhang, Liang Chen, Zhanqing Zhang, Lungen Lu, Demin Yu, Yue Han, Donghua Zhang, Peizhan Chen, Xiaonan Zhang, Zhenghong Yuan, Jinyan Huang, Xinxin Zhang

**Affiliations:** 1 Research Laboratory of Clinical Virology, Ruijin Hospital, Shanghai Jiaotong University, School of Medicine, Shanghai 200025, China; 2 Department of Infectious Diseases, Ruijin Hospital, Shanghai Jiaotong University, School of Medicine, Shanghai 200025, China; 3 Department of Infectious Diseases, Huashan Hospital, Fudan University, Shanghai 200040, China; 4 Shanghai Public Health Clinical Center, Fudan University, Shanghai 201508, China; 5 Department of Gastroenterology, Shanghai General Hospital, Shanghai Jiaotong University, School of Medicine, Shanghai 200080, China; 6 Translational Medicine Research Center, Ruijin Hospital North, Shanghai Jiao Tong University, School of Medicine, Shanghai 201821, China; 7 Key Lab of Medicine Molecular Virology of MOE/MOH, Shanghai Medical School, Fudan University, Shanghai 200032, China; 8 State Key Laboratory of Medical Genomics, Ruijin Hospital, Shanghai Jiaotong University, School of Medicine, Shanghai 200025, China

## Abstract

Although hepatitis B virus (HBV) infection is the leading cause of liver fibrosis (LF), the mechanisms underlying liver fibrotic progression remain unclear. Here, we investigated the gene expression profiles of HBV-related LF patients. Whole genome expression arrays were used to detect gene expression in liver biopsy samples from chronically HBV infected patients. Through integrative data analysis, we identified several pathways and key genes involved in the initiation and exacerbation of liver fibrosis. Weight gene co-expression analysis revealed that integrin subunit β-like 1 (ITGBL1) was a key regulator of fibrogenesis. Functional experiments demonstrated that ITGBL1 was an upstream regulator of LF via interactions with transforming growth factor β1. In summary, we investigated the gene expression profiles of HBV-related LF patients and identified a key regulator ITGBL1. Our findings provide a foundation for future studies of gene functions and promote the development of novel antifibrotic therapies.

Approximately 240 million people are chronically infected with hepatitis B virus (HBV), and more than 780,000 people die each year, owing to complications of hepatitis B, including HBV-related fibrosis, cirrhosis and hepatocellular carcinoma^1^. HBV-related mortality is especially a problem in Asia because of the higher prevalence of hepatitis B^2^.

Liver fibrosis is characterized by the perpetuation of the normal wound-healing response, thus resulting in the increased synthesis and deposition of extracellular matrix (ECM) within the injured tissue. Emerging evidence has indicated that host genetic^3^, virological^4^ and immunological factors^5^,^6^ influence liver fibrotic progression. To investigate the correlation between host/virus factors and gene expression, 124 HBV-related LF patients were enrolled in this study on the basis of their clinical traits, including histopathologic score, virological, and biochemical markers.

Fibrogenesis is a complex process involving a number of different cells, including hepatocytes, hepatic stellate cells (HSCs) and immunocytes. Activation of HSC and transdifferentiation into myofibroblast-like cells are considered the key steps in liver fibrosis^5^. HSC activation is primarily driven by fibrogenic cytokines and growth factors released by activated inflammatory cells and epithelia cells (including hepatocytes and cholangiocytes) among which TGFβ1 is the most prominent regulator^6^. Many genes participate in fibrogenesis through interactions with the TGFβ signaling pathway, among which integrins are crucial. Integrins are cellular receptors that consist of an α and a β subunit and form at least 24 different dimers that mediate cell-cell and cell-ECM interactions^7^. Studies have demonstrated that integrins play important roles in fibrogenesis^6^. During biliary fibrosis progression, integrin α vβ6 is strongly upregulated in cholangiocytes and drives fibrogenesis via TGFβ1 activation^8^.

In the present study, we focused on gene functions and gene-gene correlations on the basis of expression data from HBV-related LF patients. Several hub genes and pathways highly related to fibrotic progression were discovered, among which integrin subunit β like 1 (ITGBL1) was identified as a key regulator. The functional roles of ITGBL1 were clarified by using *in vitro* experiments, which showed that ITGBL1 promotes HSC activation and liver fibrosis by upregulating TGFβ1. These findings demonstrate a pathological role of ITGBL1 and provide essential statistical evidence for further research on HBV-related liver fibrosis which may facilitate the development of novel antifibrotic therapies.

## Results

### Global view of gene expression patterns associated with clinical traits

After removal of non-annotated, nonspecific and redundancy duplicated probe sets, 21,651 of 54,675 probe sets were filtered out. The normalized intensity values of these probes were used for unsupervised hierarchical clustering. A total of 13 clinical traits of each patient were also measured, including histological scores, liver and serum virological markers and serum biochemical markers. Liver biopsy is considered to be the “gold standard” for the diagnosis of fibrosis, and sequential histological staging of fibrosis (Scheuer score ‘S’) and grading of inflammation (Scheuer score ‘G’) can be used to assess disease progression^2^. Virological markers including HBV DNA, hepatitis B surface antigen (HBsAg), hepatitis B surface antibody (HBsAb), hepatitis B e antigen (HBeAg), and hepatitis B e antibody (HBeAb) are widely used to monitor viral activity and direct antiviral therapy. The biochemical markers alanine amino transaminase (ALT) and aspartate amino transaminase (AST) have also been widely used to assess liver inflammation and function. The clinical traits of each patient are listed in Supplementary Table S1.

**Figure 1:**
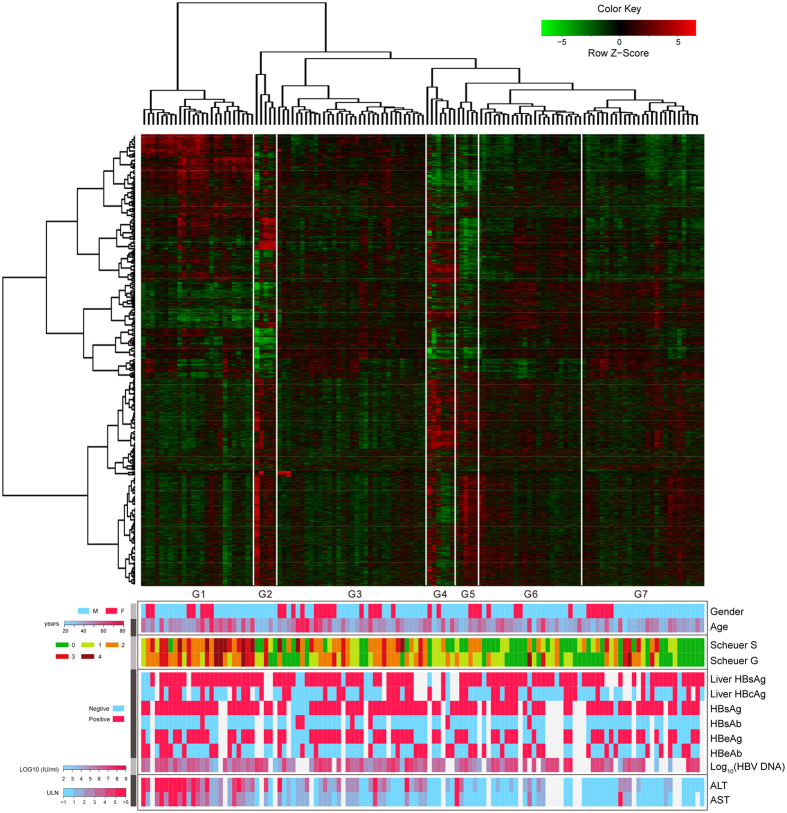
Unsupervised hierarchical clustering showed significant patterns with clinical traits. Unsupervised hierarchical clustering was performed on the basis of the expression values of 21,651 probe sets. The rows in the heatmap indicate the gene expression values and the columns indicate the 124 samples examined. The bottom color panels show the 13 clinical traits of each patient. Different types of clinical traits were divided into separated boxes. The clustering of samples and the column dendrogram showed 7 significant subgroups (SG1 to SG7). The colors corresponding to the scales bars and traits are shown on the left.

Unsupervised clustering of the gene expression in 124 HBV-related LF patients identified 7 subgroups (SG1 to SG7) on the basis of sample clustering (Fig. 1). To further investigate the relationship between clinical traits and sample clustering, Pearson’s correlation test was used to determine whether the associations between sample subgroups and clinical parameters were significant. The results showed that Scheuer score was most significantly related to sample subgrouping (*P* = 1.26 × 10^−8^ with fibrosis stages and *P* = 2.70 × 10^−7^ with inflammation grades, Pearson’s correlation test). Age, ALT and AST were also related to sample subgroups, although the significances of age (*P* = 0.01), ALT (*P* = 9.95 × 10^−5^) and AST (*P* = 1.80 × 10^−3^) were weaker than that of the Scheuer score. This result is consistent with the frequent clinical application of age and ALT/AST in FIB-4 and Forn’s index for noninvasive fibrosis prediction and diagnosis^9^. To validate the subgrouping of the samples, a principal component analysis (PCA) was performed to analyze the association between the gene expression profile and the subgroups defined by unsupervised hierarchical clustering. PCA revealed 7 distinct clusters (Supplementary Figure S1). PC1 and PC2 explained 99.99% of the total variance. The correlation significance between PC1, PC2 and subgrouping (SG1 to SG7) was measured using Pearson’s test (*P* = 2.20 × 10^−16^).

### Differential expression showed dynamic changes with disease progression

**Figure 2:**
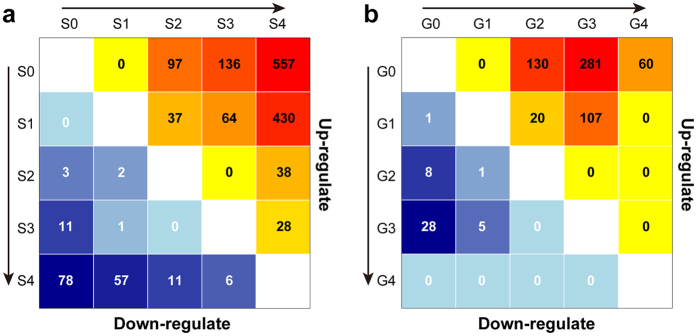
Number of DEGs among different histological stages and grades. The number of DEGs was calculated for each fibrosis stages (**a**) and inflammations grades (**b**) and are visualized using a heatmap. Upregulated genes are shown in yellow to red in the upper section, and downregulated genes are shown in lightblue to darkblue in the lower section. The intensity of the background colors corresponds to the number of DEGs.

As liver histological Scheuer Score has been demonstrated to afford the most relevant assessment of fibrosis and inflammation, it was then applied to screen for differentially expressed genes (DEGs) between different stages of liver fibrosis and inflammation. For liver fibrosis, we analyzed 43 samples in S0, 20 samples in S1, 18 samples in S2, 33 samples in S3 and 10 samples in S4. For inflammation, 37 samples in G0, 33 samples in G1, 34 samples in G2, 15 samples in G3 and 5 samples in G4 were analyzed and compared in a pairwise manner. All DEGs are listed in Supplementary Table S2. We observed that more DEGs were upregulated in advanced levels than in mild levels (Fig. 2), which may due to the activation of multiple pathways regulating cell responses to injury^2^. Interestingly, we also observed recurrently downregulated genes across stages, thus indicating their potential negative regulation of liver fibrosis. The numbers of up- and downregulated DEGs increased with histological score, except for inflammation grade 4, thus indicating a dynamic change related to disease progression. The number of DEGs related to fibrosis reached the maximum value at S4, whereas the number of DEGs related to inflammation peaked at G3.

Surprisingly, although we used accommodative filtration criteria that did not rely heavily on statistical significance (*P* value), no DEGs were screened between certain comparisons. Notably, almost no DEGs satisfied the filtering criteria when S0 was compared with S1 and G0 was compared with G1, indicating that fibrogenesis related genes were not significantly up- or downregulated in mild levels, a result consistent with clinical practices. According to several guidelines^10^,^11^, fibrosis scoring lower than S2 are regarded as “mild level”.

### ITGBL1 was highly associated with fibrosis stages

**Figure 3:**
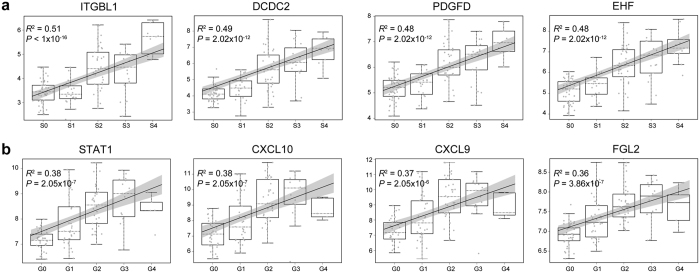
Top 4 highly related genes identified by trend test. Lineage regression models were constructed to evaluate the relationship between gene expression and histological scoring. Box plot and fitting curve with a 95% CI were drawn to illuminate the trend of expression value changes. The x-axis corresponds to different groups, and y-axis corresponds to normalized expression value. R-squared values and *P*-values of regression models were also implied. (**a**) ITGBL1, DCDC2, PDGFD and EHF were identified as the top 4 genes highly associated with fibrosis staging. (**b**) STAT1, CXCL10, CXCL9 and FGL2 were identified as the top 4 genes highly associated with inflammation grading.

Linear regression models were constructed using expression values and histological scores, from which *P*-values and R-squared values were derived. Genes with *P* < 0.01 (Benjamini-Hochberg (BH) adjusted) were considered to be highly associated with disease severity. A total of 3,279 probe sets (2,022 genes) were screened (Supplementary Table S3), among which ITGBL1 showed the most significant *P-*value (<1 × 10^−16^) and highest R-squared value (0.5131) with fibrosis stages. To validate the expression of ITGBL1 measured by microarrays, quantitative real-time polymerase chain reaction (qRT-PCR) was used to amplify and quantify the expression of ITGBL1 in HBV-related LF patients. As shown in Supplementary Figure S3, ITGBL1 expression was highly associated with Scheuer scores and the results were consistent with microarray results. The expression values and fitting curves of ITGBL1, doublecortin domain containing 2 (DCDC2), platelet derived growth factor D (PDGFD), and ETS homologous factor (EHF) which are highly associated with fibrosis stage and signal transducer and activator of transcription 1 (STAT1), C-X-C motif chemokine ligand 10 (CXCL10), C-X-C motif ligand 9 (CXCL9), fibrinogen like 2 (FGL2) which are highly correlated with inflammation grade are shown in Fig. 3. Among these top-ranked genes, the pathological roles of PDGFD, STAT1, CXCL10 and CXCL9 in liver fibrosis and inflammation have been described^12^,^13^,^14^,^15^. The inhibitory functions of FGL2, including the regulation of Tregs in CHB patients and the reduction of HCC angiogenesis, have also been reported^16^.

Interestingly, the expression of some genes was highly negatively correlated with disease severity, implying potential negative regulation effects on fibrogenesis. For example, the expression of Glycine-N-Methyltransferase (GNMT) was downregulated as fibrosis and inflammation progressed. Studies have reported the downregulation of GNMT in cirrhotic patients (from hepatitis C virus (HCV) and alcoholic steatohepatitis (ASH) etiologies)^17^ and this downregulation promotes a proinflammatory environment in the early stages of non-alcoholic steatohepatitis (NASH)^18^. On the basis of the pathogenetic mechanisms introduced by GNMT deficiency, novel anti-fibrosis compounds have been discovered^19^, thus suggesting the potential clinical value of these downregulated genes. Notably, several hormone metabolism relevant genes have been found to have a significantly negative association with liver fibrosis, including steroid 5 alpha-reductase 2 (SRD5A2), and the cytochrome P450 gene family (Supplementary Figure S2). These genes are major enzymes sequentially participating in pathways of estrogen and androgen biosynthesis and inactivation; they also participate in HCV-related liver cirrhosis, particularly the proteins in the cytochrome P450 gene family^20^,^21^.

### TGFβ signaling and epithelial mesenchymal transition (EMT) played important roles in initiating and promoting fibrotic progression

**Figure 4:**
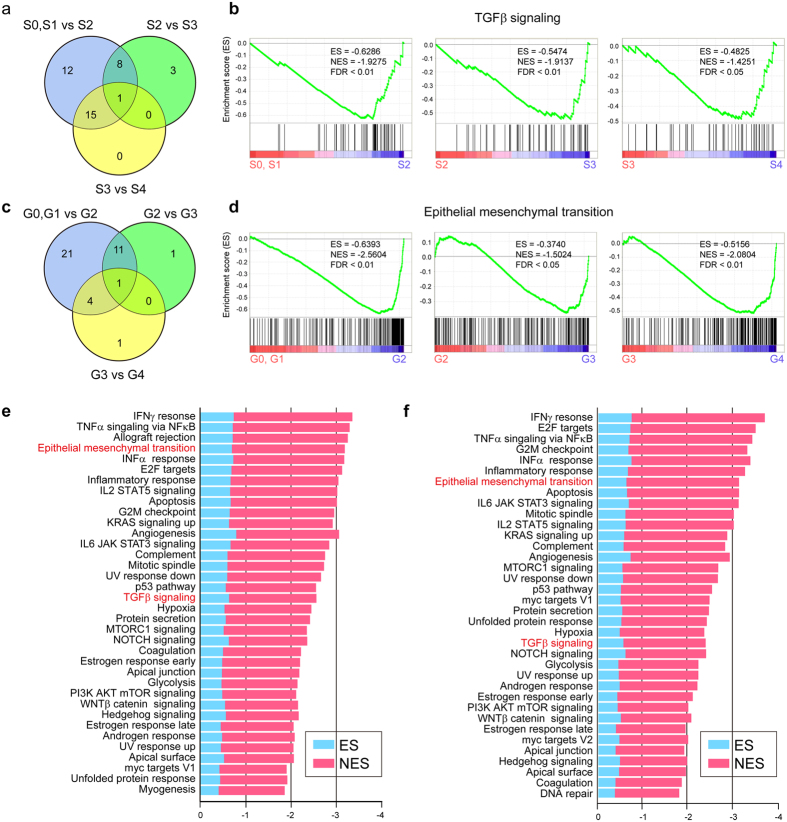
Gene sets TGFβ signaling and EMT played crucial roles in disease progression. (**a**) Venn plot showing the intersection of enriched gene sets through fibrosis progress. A total of 36, 12 and 16 gene sets were significantly enriched in comparisons between S0, S1 and S2; S2 and S3; and S3 and S4, respectively. Only TGFβ signaling was significantly enriched in all three comparisons. (**b**) Gene set TGFβ signaling mapping the details between different fibrosis stages. (**c**) Venn plot showing the intersection of enriched gene sets as inflammation progressed. A total of 37, 14 and 6 SEGSs were identified. Only one SEGS was identified in the intersection, referring to the epithelial-mesenchymal transition. (**d**) Mapping details of EMT across inflammation grades. (**e**) SEGSs identified when compared mild level fibrosis S0, S1 with S2. (**f**) SEGSs identified when comparing mild level inflammation G0 and G1 with G2. In e and f, the blue bar represents the ES, and the red bar represents the NES. EMT and TGFβ are highlighted in red.

To get further insight into the biological roles of pathways, gene set enrichment analysis (GSEA) was performed. Samples were separated into 4 levels according to the above results and previous guidelines^10^,^22^,^23^. S0 and S1 (G0 and G1) were regarded as mild levels, S2 (G2) was regarded as a moderate level, S3 (G3) was regarded as a severe level, and S4 (G4) was regarded as the end stage. Significantly enriched gene sets (SEGSs) between the two adjacent stages were screened with the filter criterion false discovery rate (FDR) <0.05. To identify the pathways participated in the initiation of the fibrotic progress, we performed GSEA based on fibrosis stages S0, S1 versus S2, and found that inflammation-relevant pathways, including the IFNγ response, TNFα signaling, and IL2 STAT5 signaling were activated and enriched with high enrichment score (ES) and normalized enrichment score (NES) (Fig. 4e). The gene sets involved in cell growth, cell proliferation, and transformation were also significantly enriched when inflammation progressed (Fig. 4f). Comparing the SEGS related to fibrosis with those related to inflammation, we observed that the majority of SEGSs were identical whether in fibrosis stages or inflammation grades. This result indicated that fibrotic and inflammation progression interact with each other, thus constituting an integrated biological process.

Next, SEGSs in different stages were intersected to highlight the crucial gene sets throughout fibrotic progression. Surprisingly, the results showed that only 1 gene set, TGFβ signaling, was significantly enriched from mild to end stage liver fibrosis (Fig. 4a,b). We applied the same SEGSs screening strategy for liver inflammation grading. Interestingly, also only a single gene set, epithelial mesenchymal transition (EMT), was significantly enriched during inflammation progression (Fig. 4c,d). HSCs play a crucial role in liver fibrosis, although the origin of these mesenchymal cells remains controversial. One hypothesis suggests bone marrow derived progenitor cells whereas another favors EMT in the local formation from hepatic epithelium. The results of present study indicated that EMT was crucial in transforming epithelial cells into activated mesenchymal cells.

### Gene modules associated with disease severity were identified by weighted gene coexpression network analysis (WGCNA)

To identity discrete groups of co-expressed genes associated with disease severity and to integrate the observed expression differences into a higher system level context, we constructed a weight gene co-expression network using normalized expression value of 21,651 selected genes (see methods of unsupervised hierarchical clustering).

The genes were grouped into 22 well-defined co-expression modules by using the TOM algorithm (Supplementary Figure S4). The modules were named after different colors according to the WGCNA conventions^24^. To validate the accuracy and conservation of these gene modules, 60 samples (validation set) were randomly selected from 122 samples (training set, 2 outliers removed) and used for module preservation simulation with 200 permutations. The results indicated that module preservation in the validation samples was highly consistent with the modules we identified in the training set (Supplementary Figure S4, Supplementary Table S4). Only module royalblue exhibited inferior preservation lower than 20, whereas the other modules, particularly modules turquoise, red and pink exhibited Z summaries higher than 40.

Co-expression networks facilitate the analysis of gene expression variations associated with multiple disease-related traits. We assessed the module eigengene relationship with clinical traits of each patient, thus providing a complementary assessment of these potential confounders (Supplementary Figure S5). Notably, modules turquoise and darkgreen were positively associated with Scheuer scoring whereas modules pink and red were negatively related. These four modules were also associated with ALT/AST but not any of the other potential confounding variables.

To further investigate the relationship between gene expression and fibrosis stages and to validate the confidence of trait-significant modules, a Student’s t test (two sides, *P*-value adjusted by BH procedure) was performed. The results showed highly similar clustering patterns corresponding to trait-significant modules turquoise, pink, red and darkgreen. Interestingly, the gene expression patterns at S0 and S1 were highly different from those observed at S2, S3 and S4. As shown in Supplementary Figure S4b, the genes corresponding to module turquoise were negatively enriched in S0 and S1, whereas a positive association with S2, S3 and S4 was also observed. This result provided direct genetic evidence supporting clinical practices in which S1 is regarded as indicating a mild level similar to S0.

**Figure 5:**
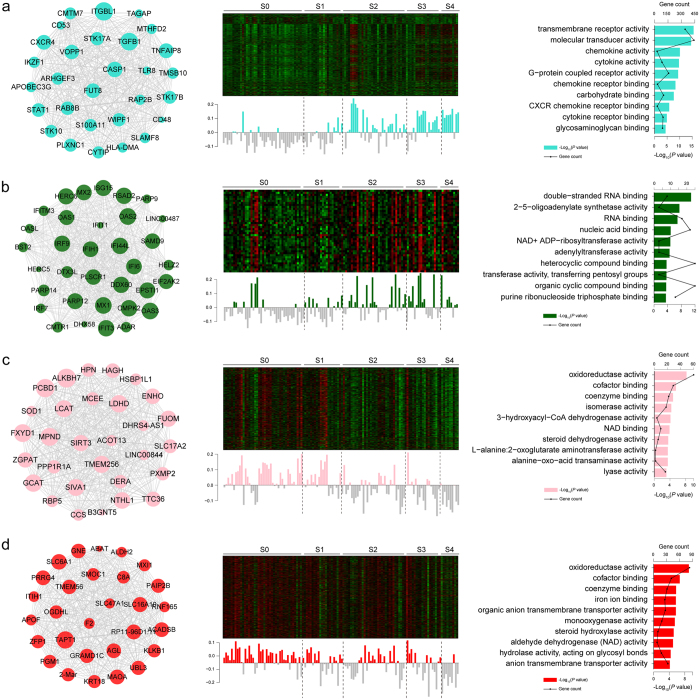
Significant modules identified by using WGCNA. The network of hub genes, eigengene expression and GO enrichment of the modules turquoise (**a**), darkgreen (**b**), pink (**c**) and red (**d**) are shown. In the network, node size corresponds to intramodular connectivity values (kWithin). Eigengene expression less than 0 is shown as gray bars, and bars with values more than 0 are filled with the color of the module. The samples are divided into 5 groups by dotted lines on the basis of fibrosis stage. The bars in the GO enrichment results represent the −log_10_ (*P-*value) and the lines represent the gene counts within a GO term.

The expression levels of each module were summarized according to the first principal component (referred to the module eigengene), and these results were used to assess whether the modules were associated with clinical phenotypes. Modules turquoise and darkgreen exhibited elevated expression in advanced fibrosis, whereas modules pink and red exhibited higher expression in mild fibrosis (Fig. 5).

The biological characteristics of the identified modules were tested using the existing data on protein-protein interactions in the STRING database. Twenty out of the 22 modules showed significant enrichment in interactions (*P* < 0.01), thus suggesting that the modules identified in the present study are biologically relevant (Supplementary Table S5). Additionally, trait-significant modules showed the highest average node degree (AND), particularly modules turquoise (AND = 17.5) and darkgreen (AND = 13.3).

Gene ontology (GO) enrichment of trait-significant modules were performed to further investigate the gene functions. The majority of the genes in the turquoise module were enriched for GO categories related to signal transduction and chemokine, and cytokine activity. The genes in darkgreen module were primarily related to genetic molecular synthesis and modification (Fig. 5a,b). Notably, the majority of the GO terms enriched in modules pink and red were related to physiochemical metabolism and steroid dehydrogenase activity (Fig. 5c,d), a result consistent with above results in the trend test.

### ITGBL1 was identified as a key regulator of fibrogenesis

A further advantage of WGCNA is that this technique enables the identification of hub genes on the basis of intramodular connectivity and network topological structure^24^. To identify hub genes with high confidence, multiple algorithms were measured to evaluate the hub score of each gene within the relevant modules (Supplementary Table S6). Among all the modules, the turquoise module showed a highly significant association with fibrosis and inflammation levels (*P* = 1 × 10^−09^ for fibrosis stages and *P* = 1 × 10^−10^ for inflammation grades). As shown in Fig. 5a, the hub genes with the highest rank of the turquoise module were ITGBL1, TGFβ1, C-X-C motif chemokine receptor 4 (CXCR4), and STAT1, among which ITGBL1 was identified as a key regulator with the highest intramodular connectivity (kWithin equal to 0.9499). TGFβ1, STAT1, and CXCR4^6^,^13^,^25^ have previously been implicated in liver fibrosis. The hub gene of additional modules highly associated with fibrosis stages, including modules darkgreen, pink and red are shown in Fig. 5b,c and d. To further explore the function of ITGBL1 in non HBV-related LF patients, the expression of ITGBL1 in 12 LF patients with ASH, NASH or chronic HCV infection were measured. As shown in Supplementary Figure S6, the expression of ITGBL1 showed none significant statistical difference between HBV-related and non HBV-related LF patients. This result indicated that ITGBL1 was not a specific regulator in HBV-related LF patients, it might also be crucial in non HBV-related LF patients.

**Figure 6:**
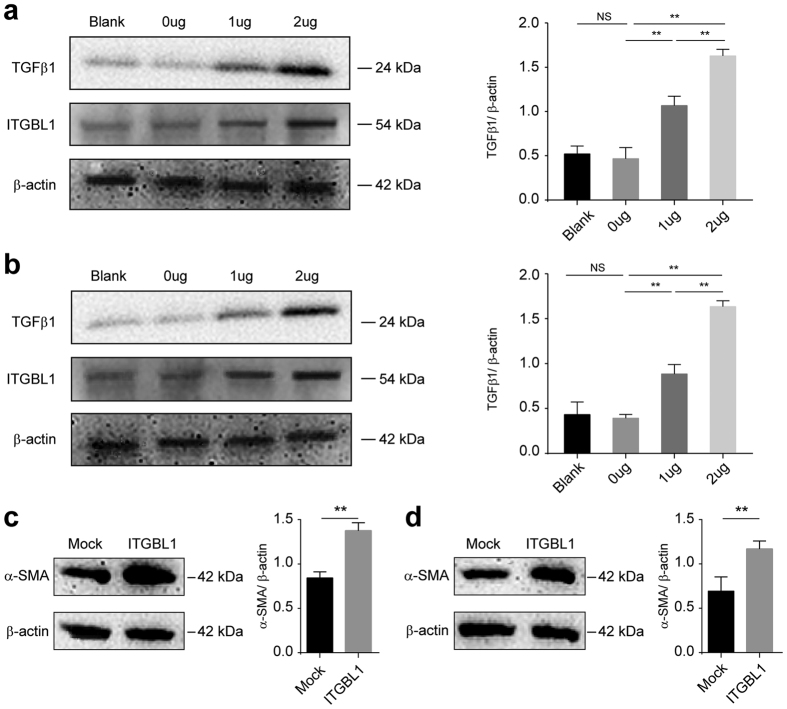
ITGBL1 upregulates expression of TGFβ1 and promotes HSC activation. Western blotting of TGFβ1, ITGBL1 and β-actin in (**a**) Huh7 cell line and (**b**) HepG2 cell line. Transfection reagent without plasmids was used as a blank. 0 μg: 0 μg of ITGBL1 and 2 μg of Mock; 1 μg: 1 μg of ITGBL1 and 1 μg of Mock; 2 μg: 2 μg of ITGBL1 and 0 ug of Mock. Western blotting of α-SMA and β-action in LX-2 cells treated with condition medium from (**c**) Huh7 cell line and (**d**) HepG2 cell line. Mock: Condition medium from hepatocytes transfected with Mock plasmids; ITGBL1: Condition medium from hepatocytes transfected with ITGBL1 cDNA expression plasmids (n = 3, **P < 0.01, NS not significant, Student’s *t*-test).

Studies have previously that ITGBL1 is an upstream activator of the TGFβ signaling pathway in breast cancer bone metastasis and promotes cell migration and adhesion though interactions with TGFβ1^26^. To determine whether ITGBL1 plays a key role in fibrogenesis and to examine its interaction with TGFβ1, cDNA expression plasmid of ITGBL1 was transfected into Huh7 and HepG2 cell lines. The protein expression detected by western blotting is shown in Fig. 6. The expression of TGFβ1 was significantly upregulated in Huh7 cells with an increasing amount of transfected ITGBL1 plasmid, as shown in Fig. 6a (P < 0.01), and the same effect was observed in HepG2 cells (Fig. 6b). To further demonstrate the effect of ITGBL1 on HSC activation and ECM deposition, human stellate cell line LX-2 was used and treated with condition medium from hepatocytes transfected with ITGBL1 expression plasmid. The result showed that elevated ITGBL1 significantly up-regulated α-SMA expression (Fig. 6c,d, *P* < 0.01) and promoted HSC activation.

## Discussion

Viral hepatitis is the dominant etiology of liver fibrosis. However, the gene expression profile of HBV-related liver fibrosis based on a large cohort of patients has not been studied yet. In the present study, 124 treatment naive patients were enrolled from multiple centers, and liver biopsy samples with detailed clinical traits were collected. Fibrosis is considered a complex biological process involving extensive genes and pathways. To investigate gene functions and gene-gene correlations, the expression profile of each patient was obtained.

We performed unsupervised hierarchical clustering and PCA to determine the relationship of various host/virus factors with fibrotic progression. The associations between clinical traits and expression profiles indicated that gene expression in chronically HBV infected LF patients was highly correlated with pathological Scheuer scores. Although biopsy is prone to considerable sampling variability^27^,^28^, it still shows higher specificity than the other markers. Subsequent analyses were then performed on the basis of Scheuer scores, including differential expression analysis. Notably, patients with inflammation grade 4 (G4) showed no significant up- or downregulated DEGs compared with other grades, a result probably reflecting the specific T cell exhaustion in adaptive immunity toward HBV infection^29^.

Although liver biopsy is a reliable standard for detecting histological change and guiding clinical therapy, the invasiveness and high risk of this technique for coagulopathy impediment patients remain problematic. Several indices, such as APRI, FIB-4, GPR, RPR, and Forn’s index, have previously been developed for noninvasive diagnosis; however, the performances of these indices differs. Thus, novel predictive models should be developed. Liver injury consistently accompanies continuous cell apoptosis and necrosis; subsequently, cellular components are released from disrupted cells, thereby producing relevant gene products detectable in patient sera. We screened 2,022 genes whose expression was highly correlated with disease severity by using trend test. With the rapid development of liquid biopsy and other sensitive technologies, these genes may be used as promising biomarker candidates.

The results of differential expression analyses and trend tests revealed that anti-fibrotic mechanisms played important roles in fibrosis progression. STAT1 is an important negative regulator in liver fibrosis. The activation of STAT1 attenuates liver fibrosis through the inhibition of HSC proliferation, attenuation of TGFβ signaling, and stimulation of the NK cell killing of HSCs^13^. STAT1 expression was upregulated with fibrosis progression, thus indicating the existence of certain active endogenous anti-fibrosis mechanisms in hepatic cells. Another gene procollagen C-Endopeptidase enhancer 2 (PCOLCE2) shows lower expression in advanced stages of fibrosis and inflammation than in early stage. PCOLCE2 encodes a functional procollagen C-proteinase enhancer^30^ and participates in the degradation of the extracellular matrix pathway. In general, genes with anti-fibrotic functions are simultaneously activated after the initiation of fibrosis progression to protect host from advanced injury. The function of these genes should be further investigated, because they might play crucial roles in maintaining host fibrosis/anti-fibrosis equilibrium homeostasis under physiological circumstances or otherwise promote fibrotic progression under pathological conditions.

A WGCNA was performed to identify trait-significant modules and hub genes. Module lightyellow was considered as the “inner control” to demonstrate the accuracy of module identification and hub gene screening. Module lightyellow was extremely highly associated with gender (*P*-value = 2 × 10^−49^, correlation equal to 0.92). Several aspects of this module were investigated. First, the genome location of each gene within module lightyellow was retrieved. Nearly half of the genes (28/61) were located in chromosome X or Y. Second, GO analysis of this module showed that chromatin modification and chromosome organization were the top-ranked GO terms. Third, among the hub genes screened, two genes (XIST and TSIX) were well-known non-coding RNAs relevant to chromosome X inactivation^31^,^32^.

ITGBL1 was identified as the highest ranked hub gene related to fibrosis stage by WGCNA and its interaction with TGFβ1 in hepatocytes was demonstrated by using *in vitro* experiments. The GSEA results identified the TGFβ signaling pathway as a key regulator of fibrogenesis. Therefore, elevated expression of ITGBL1 suggests that hepatocytes might activate the TGFβ signaling pathway by upregulating ITGBL1 and subsequently promoting HSC activation in HBV-related LF patients.

There were some inevitable limitations of the present study. For patient enrollment, the patients at different fibrosis stages were not equivalent and more patients at advanced fibrosis stages were needed. With the emergence and extensive application of noninvasive diagnostic technologies, such as FibroScan, the use of liver biopsy for fibrosis diagnosis is decreasing rapidly, thus making it difficult to collect liver biopsy samples. In addition, severe liver fibrosis and inflammation are consistently accompanied by decompensated liver dysfunctions, so percutaneous puncture into the liver might cause severe visceral hemorrhage. Second, additional *in vivo* and *in vitro* experiments are needed to further study the functions of the hub genes.

In conclusion, in the present study, we investigated the whole genome expression profiles of HBV-related liver fibrosis patients and determined the association of gene expression patterns with different clinical traits. Several pathways, including the TGFβ signaling and epithelial mesenchymal transition were demonstrated to play crucial roles in fibrotic progression. Significantly related genes were screened that may potentially be used as biomarkers of hepatic pathogenesis and therapeutic targets for anti-fibrosis therapies. Among the associated genes, we also identified ITGBL1 as a hub gene in fibrogenesis and clarified its functional role with TGFβ1.

## Methods

### Study subjects

Liver biopsy samples from 136 chronically HBV infected patients were obtained. Among the recruited patients, 124 patients were admitted to Shanghai Ruijin Hospital, Shanghai Huashan Hospital, Shanghai Public Health Clinical Center and Shanghai General Hospital between 2009 and 2011, and 12 patients were admitted to Shanghai Ruijin Hospital in 2016. Liver biopsy samples from 12 non HBV-related LF patients admitted to Shanghai Ruijin Hospital were enrolled, including 5 patients with chronic HCV infection, 4 patients with NASH and 3 patients with ASH. All patients were diagnosed on the basis of the criteria recommended by the Asian Pacific Association for the Study of the Liver (APASL)^33^ and had not taken any antiviral therapies or immunosuppressive drugs within six months before sampling. Among HBV infected patients, individuals with concurrent hepatitis C virus, hepatitis D virus or human immunodeficiency virus infection, autoimmune liver disease, drug induced liver injury, alcoholic liver disease or hepatocellular carcinoma were excluded. All the samples were send to the Pathology Department of Shanghai Fudan University, School of Medicine for histopathology diagnosis. Two experienced pathologists independently measured and confirmed the fibrosis stating (Scheuer S) and inflammation grading (Scheuer G)^34^,^35^. Written informed consent was obtained from each subject. The study protocol was approved by the ethics committee of Ruijin Hospital, Shanghai Jiaotong University, School of Medicine and all the methods were carried out in accordance with the approved guidelines.

### RNA extraction and microarrays

Total RNA was extracted from approximately 100 mg of frozen tissue, using the RNEasy Mini kit (Qiagen, Germantown, Maryland, USA) according to the manufacturer’s instructions. Nanodrop 2000 spectrophotometer (Thermo scientific, Wilmington, Delaware, USA) was used to measure the RNA concentration, Agilent 2100 Bioanalyzer (Agilent, Santa Clara, California, USA) was used to determine RNA purity/integrity. cDNA synthesis, labeling, and hybridizations were performed on Affymetrix Human Genome U133 Plus 2.0 arrays (Affymetrix, Cleveland, Ohio, USA), and staining and scanning were performed according to the manufacturer’s instructions.

### qRT-PCR

1 μg of total RNA from each sample was subjected to reverse transcription using PrimerScript RT reagent kit (Takara, Japan), according to the manufacture’s specifications. Equal amount of complementary DNA (cDNA) was used to perform real-time PCR with SYBR Premix Ex Tag Kit (Takara, Japan). Expression levels were normalized to that of β-actin and calculated using the 2^−ΔCT^ formula. Sequences of the primers used for ITGBL1 were: forward primer: 5′-TCATCTGCTCTAATGCAGGTACA-3′ and reverse primer 5′-GTTTCCACAGTAACACTTCCCA-3′; the primers used for β-actin were: forward: 5′-AGAGCTACGAGCTGCCTGAC-3′ and reverse primer 5′-AGCACTGTGTTGGCGTACAG-3′.

### Data preprocessing

To ensure the highest possible level of data quality, rigorous quality control procedures were implemented on the microarray datasets before expression values were generated. Microarray data processing was performed using the R 3.1.3 and Bioconductor packages. First, raw CEL files were normalized and background adjusted using the Robust Multichip Average (RMA) algorithm with the affy package^36^. Then, k-nearest neighbor (KNN) algorithm within impute package was applied for missing value imputation. Batch effects in the expression data were adjusted using empirical Bayes methods in the R package sva according to previous studies^37^.

### Unsupervised hierarchical clustering

The normalized expression microarray data for each patient were collected. Subsequently, the following processing steps were applied: (1) probe sets without gene annotation were omitted; (2) probes targeting multiple genes were omitted; (3) the “best” probe for each gene which was targeted by multiple probe sets was selected. Specifically, only the probe set with the maximum variance was retained when there were multiple probe sets for a gene. Subsequently, unsupervised hierarchical clustering was performed with the ward2 method in the R gplots package.

### PCA

PCA was performed using the “princomp” function in R software. The component loadings were calculated as correlations between measured expression values and principal component scores. The top 3 principal components PC1, PC2 and PC3 were visualized using 3-dimensional scatter plots by using the scatterplot3d package.

### Differential expression

Differential expression was assessed using the linear model for microarray data (LIMMA) package, a modified t-test that incorporates the BH multiple hypotheses correction method^38^. To improve the reliability of differential expressed genes, probe sets of which the adjusted *P* < 0.1 and fold-changes >2 between two comparison groups were defined as significant differentially expressed probe sets, according to the MicroArray Quality Control (MAQC) project recommendations^39^.

### GO enrichment analysis

Functional enrichment analysis was assessed using the DAVID database^40^ (http://david.abcc.ncifcrf.gov/). For differentially expressed genes and coexpression modules, the background was set to the total list of genes expressed in the human dataset. Owing to the limits of uploaded genes in DAVID, R package topGO and clusterProfile were used for GO enrichment analysis for gene lists containing more than 3,000 genes. The statistical significance threshold level for all GO enrichment analyses was *P* < 0.01 (BH corrected for multiple comparisons).

### Trend test analysis

To find the genes highly correlated with fibrosis and inflammation progression, we used trend tests to analyze the relationship between disease severity and gene expression levels. A linear model based on liver pathology Scheuer scoring and gene expression values was constructed, and the significance value was obtained and adjusted by using BH correction. R software 3.1.3 was used for data analysis and visualization.

### GSEA

GSEA was performed using GSEA software^41^ on probe set normalized data. Predefined gene sets were downloaded from the GSEA Molecular Signatures Database (http://software.broadinstitute.org/gsea/msigdb/index.jsp). Gene sets significantly over- or under-represented were returned by GSEA as showing an ES < 0, *P* < 0.05 and FDR < 0.05 when using 1,000 permutations of gene-set labels.

### WGCNA

A weighted gene coexpression network was constructed to identify the gene groups (modules) involved in liver inflammation and fibrotic progression according to a previously described algorithm^24^. The R packages WGCNA^42^ was applied to for this analysis. Briefly, the genes were screened on the basis of specific filter criteria (mentioned in the unsupervised hierarchical clustering section). Person’s correlation coefficients were calculated for all pairwise comparisons of the selected genes yielding a similarity (correlation) matrix (s_ij_). The resulting Person’s correlation matrix (s_ij_) was transformed into an adjacency matrix A = [a_ij_] using a power function, a_ij_ = Power (s_ij_, β) ≡ |s_ij_|^β^, where a_ij_ is the strength of a connection between two nodes (genes) *i* and *j* in the network. The parameter β was selected using scale-free topology criteria. Subsequently, we used average linkage hierarchical clustering to identify modules of densely interconnected genes on the basis of the topological overlap dissimilarity matrix (1 − topological overlap) of their network connection strengths^24^. Genes that were not assigned to specific modules were assigned the color grey.

### Module preservation and Student’s t-test statistics

To validate the preservation of gene modules identified by WGCNA, a validation dataset was constructed using 60 randomly selected samples. Module preservation was calculated as previously described^43^. Briefly, we used the R function “modulePreservation” in the R WGCNA package, because this quantitative measure of module preservation provides rigorous evidence that a module is not preserved. The number of permutations was set to 200. By averaging the preservation statistics through permutations, a Z summary value was calculated. The values of the module preservation Z summary statistic and median rank are shown in supplementary Table S4. To visualize the region-enrichment of each gene at different fibrosis stage, the t statistic of the samples at a given stage compared with the other samples not in this stage was determined using Student’s t tests, as previously described^44^.

### STRING biological interaction validation

The functional protein association networks database STRING v10 (http://string-db.org/)^45^ was used to identify biological interactions within the WGCNA modules. The input options were set to include ‘Textmining, ‘Experiments’, ‘Databases’, ‘Co-expression’, ‘Neighborhood’ and ‘Co-occurrence’ with an interaction confidence score more than 0.4. Built-in algorithms provided by the STRING database were applied to calculate the enrichment in intersections for each module. If the number of genes reached the upper limit of STRING, then 2,000 genes were randomly selected from the module for 10 times and the evaluating indices of modules were regarded as the average values of the random selection repeats.

### Hub gene identification and visualization

After co-expression network construction, centrality analysis was conducted using the CytoHubba^46^ plug-in in Cytoscape^47^ 2.8.2 to identify hub genes. We useed twelve built-in centrality indices—Maximal Clique Centrality (MCC), Density of Maximum Neighborhood Component (DMNC), Maximum Neighborhood Component (MNC), Degree, Edge Percolated Component (EPC), Bottleneck, Eccentricity, Closeness, Radiability, Betweenness, Stress and Clustering Coefficient to calculate the hub scores of significant modules. In addition, the intramodular connectivity (kWithin) of each gene was calculated within the WGCNA package. KWithin was obtained by summing the connection strengths (adjacencies) with other module genes and divided as the maximum intramodular connectivity in a given module. The top 30 genes ranked by each centrality index were considered to be highly ranked hub genes candidates. Among the 13 lists of potential hub genes ranked by different hub scores, we applied a strict filter criterion that only genes within the intersection of ≥6 lists were considered to be high-confidence hub genes with potential biological significance. Cytoscape formatted files were generated by R package WGCNA and visualized using Cytoscape 3.4.1.

### Cell experiments

Human hepatocellular carcinoma cell lines Huh7 and HepG2 and human HSC cell line LX-2 were cultured in Williams E medium supplemented with 1% MEM Non-Essential Amino Acids, 1 mM Sodium Pyruvate, 1% GlutaMAX, 10% fetal bovine serum (FBS) (Gibco, Life Technologies, Carlsbad, CA, USA), 100 IU/ml penicillin and 100 μg/ml streptomycin (Invitrogen, Bern, Switzerland) and maintained at 37 °C in a 5% CO_2_ humidified atmosphere. To evaluate the regulation effect of ITGBL1 on TGFβ1 expression, hepatocytes (2 × 10^5^ per well) cultured for 24 h were transfected with 2 μg of ITGBL1 cDNA expression plasmid mimic (ITGBL1 mimic Mock plasmid) using X-tremeGene 9 DNA transfection reagent (Roche, Mannheim, Germany) according to the manufacturer’s instructions. To collect condition medium for LX-2 culture, Huh7 and HepG2 were cultured in FBS free Williams E medium for 12 h after plasmid transfection. LX-2 cells (2 × 10^5^ per well) were then cultured in hepatocytes cell supernatant for 12 h before harvest.

### Western blotting

The cells were harvested at 48 h after plasmid transfection and lysed on ice for 20 min in RIPA lysis buffer (Beyotime, Nanjing, China) containing 1% protease inhibitor cocktail and 1% phosphatase inhibitor cocktail (Roche, Mannheim, Germany). The lysate was centrifuged at 12,000 g for 15 min at 4 °C, and the supernatants were subsequently collected. A BCA protein assay kit (Beyotime, Nanjing, China) was used to determine the protein concentrations. The protein samples were denatured for 10 min at 100 °C under non-reducing conditions. An equal amount of 20 μg of protein from each sample was loaded and separated by SDS-PAGE using 4% to 20% precast gradient gels (Tanon, Shanghai, China); this was followed by transfer onto polyvinylidene di-fluoride membranes (Merck Millipore, Billerica, Massachusetts, USA). After being blocked with TBST buffer containing 5% skimmed milk, the membranes were incubated with the primary antibodies against TGFβ1 (1:1,000, R&D, Minneapolis, Minnesota, USA), ITGBL1 (1:500, Abcam, Cambridge, Massachusetts, USA), α-SMA (1:1000, Sigma-Aldrich, Munich, Germany) and β-actin (1:2,000, Sigma-Aldrich, Munich, Germany) in 5% skimmed milk TBST buffer overnight at 4 °C. After being washed in TBST, the membranes were incubated with horseradish peroxidase (HRP)-conjugated secondary antibodies (1:1,000, Beyotime, Nanjing, China) at room temperature for 2 h. The bands were detected using a high-sensitivity chemiluminescent substrate ECL kit (Tanon, Shanghai, China). Western blots were quantified using ImageJ software (version 1.50).

### Data availability

The microarray data have been deposited in NCBI GEO database under the accession code GSE84044.

### Code availability

R codes used for data analyses and visualization are available upon request by contact with Prof. Xinxin Zhang.

## Additional Information

**How to cite this article:** Wang, M. *et al*. Characterization of gene expression profiles in HBV-related liver fibrosis patients and identification of ITGBL1 as a key regulator of fibrogenesis. *Sci. Rep.* **7**, 43446; doi: 10.1038/srep43446 (2017).

**Publisher's note:** Springer Nature remains neutral with regard to jurisdictional claims in published maps and institutional affiliations.

## Supplementary Material

Supplementary Information

Supplementary tables
