# Supplementary material for: Characterization of gene expression profiles in HBV-related liver fibrosis patients and identification of ITGBL1 as a key regulator of fibrogenesis
Source: Sci Rep. 2017 Mar 6;7:43446. doi: 10.1038/srep43446 (PMC5337978; doi:10.1038/srep43446)
Supplement: Supplementary Information [file srep43446-s1.pdf]

Characterization of gene expression profiles in HBV-related liver fibrosis patients and identification of ITGBL1 as a key regulator of fibrogenesis

Mingjie Wang[1], Qiming Gong[2], Jiming Zhang[3], Liang Chen[4], Zhanqing Zhang[4], Lungen Lu[5], Demin Yu[1], Yue Han[1], Donghua Zhang[1], Peizhan Chen[6], Xiaonan Zhang[4], Zhenghong Yuan[7#], Jinyan Huang[8#], Xinxin Zhang[1,6#]

1. Research Laboratory of Clinical Virology, Ruijin Hospital, Shanghai Jiaotong University, School of Medicine, Shanghai 200025, China

2. Department of Infectious Diseases, Ruijin Hospital, Shanghai Jiaotong University, School of Medicine, Shanghai 200025, China

3. Department of Infectious Diseases, Huashan Hospital, Fudan University, Shanghai 200040, China

4. Shanghai Public Health Clinical Center, Fudan University, Shanghai 201508, China

5. Department of Gastroenterology, Shanghai General Hospital, Shanghai Jiaotong University, School of Medicine

6. Translational Medicine Research Center, Ruijin Hospital North, Shanghai Jiao Tong University, School of Medicine, Shanghai 201821, China

7. Key Lab of Medicine Molecular Virology of MOE/MOH, Shanghai Medical School, Fudan University, Shanghai 200032, China

8. State Key Laboratory of Medical Genomics, Ruijin Hospital, Shanghai Jiaotong University, School of Medicine, Shanghai 200025, China

[#] Correspondence to X.Z. [zhangx@shsmu.edu.cn], J.H. [jinyan@shsmu.edu.cn] and Z.Y. [zhyuan@shaphc.org].

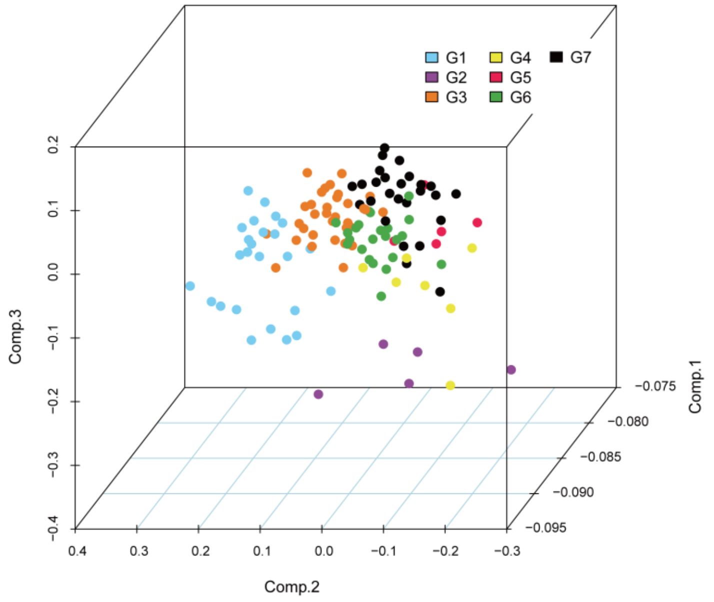

Figure S1 | Principal component analysis of gene expression

Principal component analysis of gene expression data in 124 samples, showing significant clustering among different subgroups. PC1, PC2 and PC3 were used as x-axis, y-axis and z-axis in three dimensions, respectively. Each dot delineates a sample, and the colors indicate different groups.

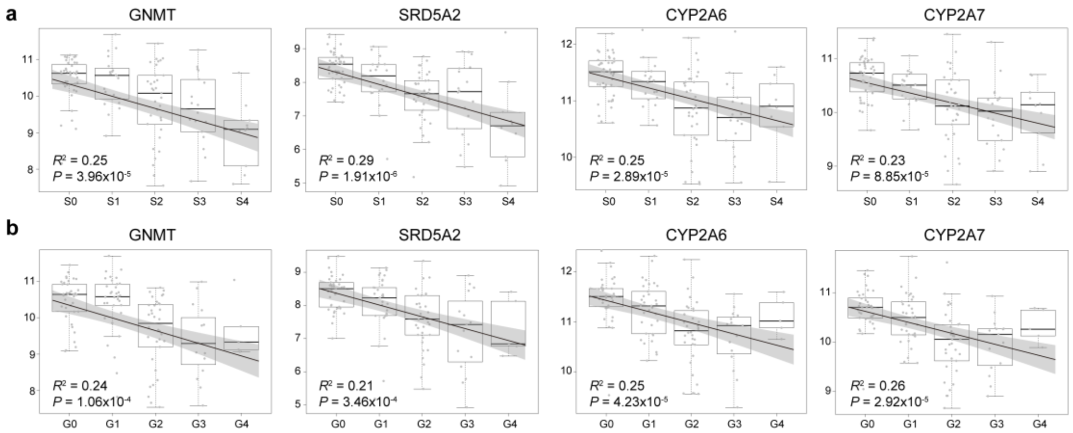

Figure S2 | Genes negatively associated with disease severity

Trend tests were used to evaluate the relationship between gene expression and histological scoring. Four negatively related genes are shown as a demonstration. The x-axis corresponds to different groups, and y-axis corresponds to normalized expression value. A box plot and fitting curve with a 95% CI were drawn to illuminate the decreasing trend of expression values. R squared values and *P*-values of regression models were implied. (a) Association between GNMT, SRD5A2, CYP2A6, and CYP2A7 expression and fibrosis stages (b) Association between GNMT, SRD5A2, CYP2A6, and CYP2A7 expression and inflammation grades.

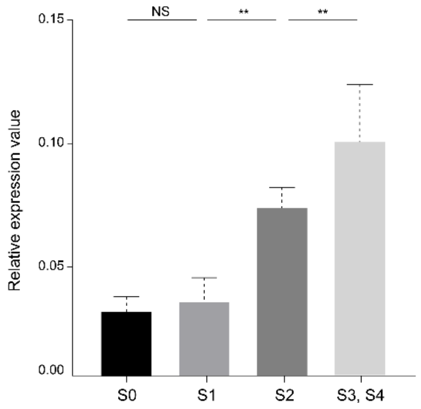

Figure S3 | Expression of ITGBL1 measured by qRT-PCR

Quantitative real-time PCR were performed by using relative quantification of mRNA level of ITGBL1 and β-actin. The relative expression levels were calculated using the $2^{-\Delta CT}$ formula. Data are represented as mean ± SEM from three independent experiments (n=3, ** $P < 0.01$, NS not significant, Student's *t*-test).

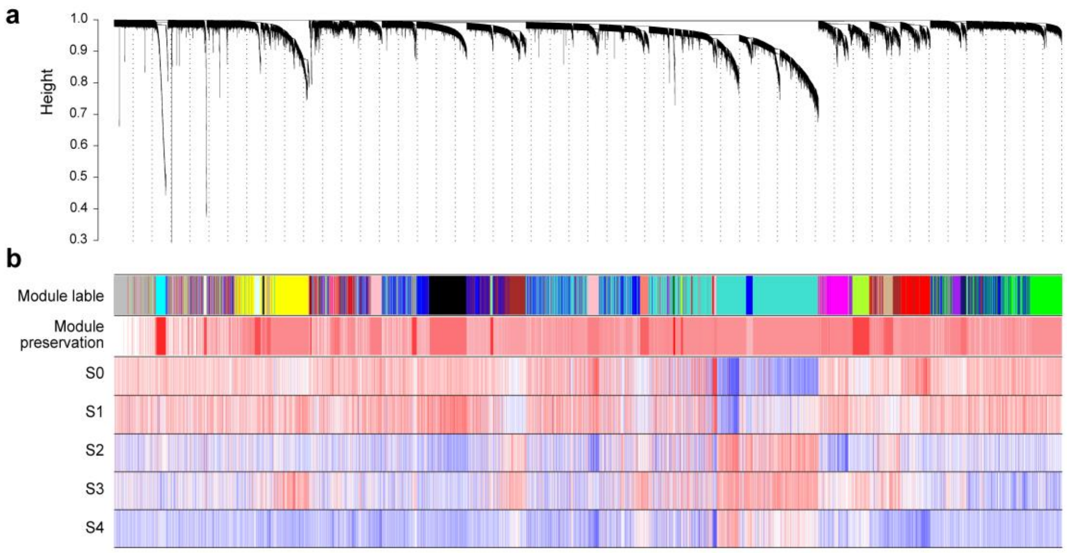

Figure S4 | Global gene networks.

(a) Cluster dendrogram grouping genes into distinct modules using 122 samples (2 outliers were removed). The y-axis corresponds to the co-expression distance between genes, and the x-axis corresponds to the genes. (b) Top color band: color-coded gene modules. Second band: Preservation of modules between the training set and validation set (defined in Methods), measured using a Z score summary. Third to sixth bands: Highly positively correlated (red) or negatively correlated (blue) genes for the particular stage. Color intensity corresponded to t statistics.

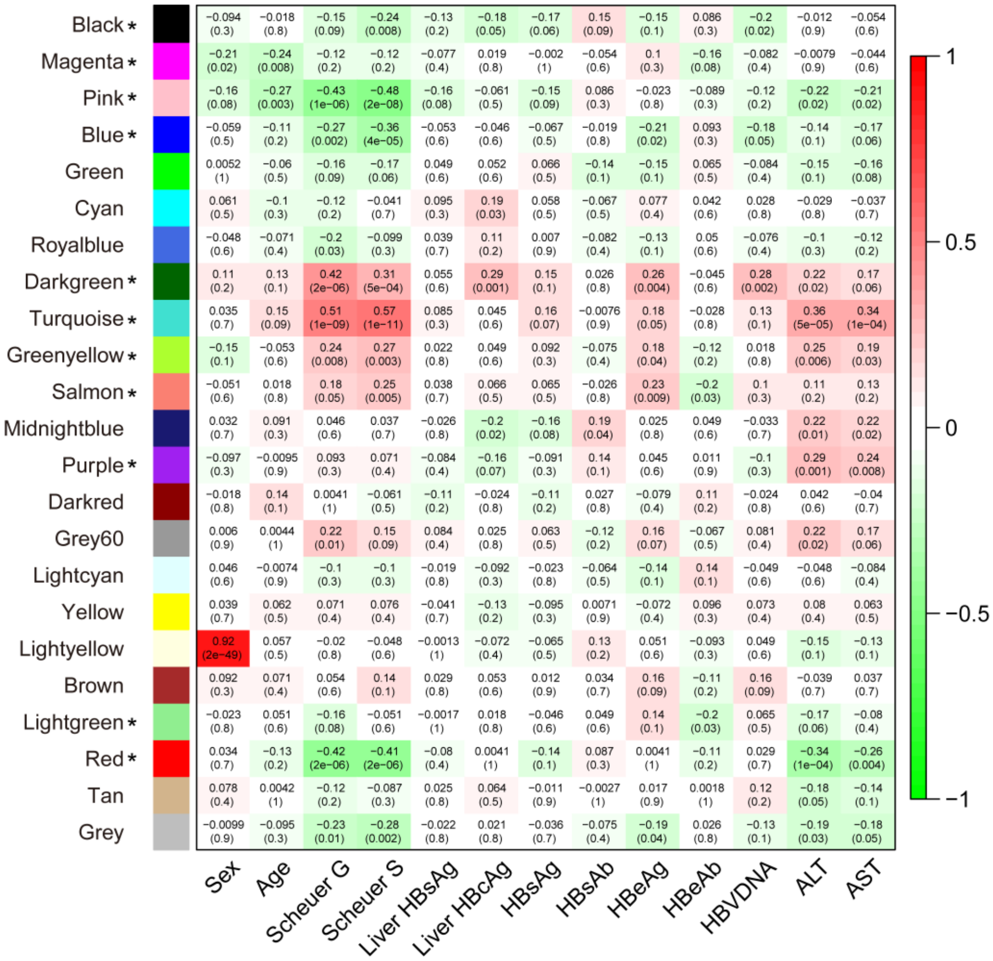

Figure S5 | Relationship between modules and clinical traits.

Heatmap of the relationship and significance between modules and clinical traits in WGCNA. The color scale bar is shown on the right. Green represents negatively related, and red represents positively related. Correlations and *P*-values were also shown in each cell. Modules with statistical significance (*P*-value < 0.05) are indicated with an asterisk.

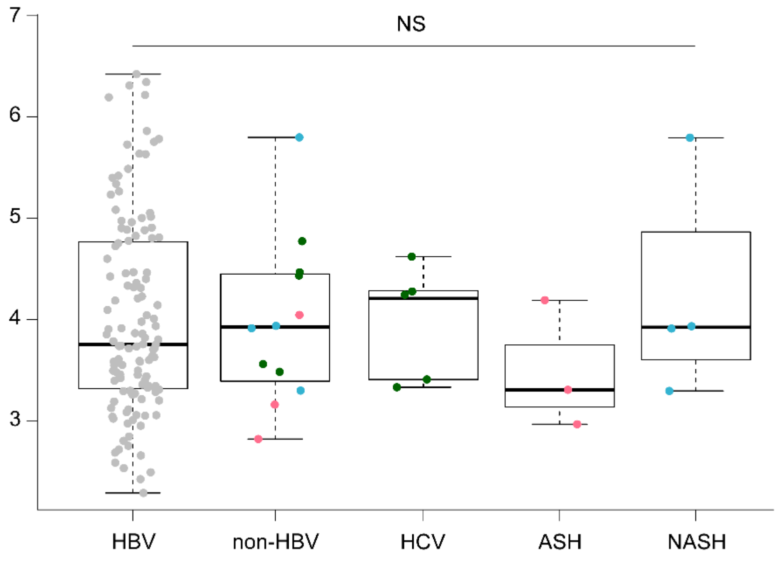

Figure S6 | ITGBL1 expression in HBV-related and non HBV-related LF patients

ITGBL1 expression in LF patients from different etiologies, including chronic HBV infection, chronic HCV infection, ASH and NASH were measured. The x-axis corresponds to different groups and y-axis corresponds to normalized expression value. Patients in HBV (n=81), HCV (n=5), ASH (n=3) and NASH (n=4) group are delineated by grey, green, pink and blue dots. Group non-HBV includes patients in HCV group, ASH group and NASH group. (NS not significant, Student's t-test).
